# Salivary gland organoid transplantation as a therapeutic option for radiation-induced xerostomia

**DOI:** 10.1186/s13287-024-03833-x

**Published:** 2024-08-26

**Authors:** Seong Gyeong Jeon, Jaeseon Lee, Su Jeong Lee, Jaehwi Seo, Jinkyoung Choi, Dong Hyuck Bae, Duk-Hee Chun, Seung Young Ko, Hyun Soo Shin, Lina Joo, Sang-Hyuk Lee, Young Chang Lim, Woo Hee Choi, Jongman Yoo

**Affiliations:** 1 R&D Institute, ORGANOIDSCIENCES Co., Ltd, 331, Pangyo-ro, Seongnam-si, 13488 Gyeonggi-do Republic of Korea; 2 Department of Microbiology, CHA University School of Medicine, 335, Pangyo-ro, Seongnam-si, 13488 Gyeonggi-do Republic of Korea; 3 Department of Anesthesiology and Pain Medicine, CHA Bundang Medical Center, CHA University School of Medicine, 59 Yatap-ro, Bundang-gu, Seongnam, 13496 Republic of Korea; 4 Department of Radiation Oncology, CHA Bundang Medical Center, CHA University, Seongnam, 13496 Republic of Korea; 5 Department of Otorhinolaryngology-Head and Neck Surgery, Kangbuk Samsung Hospital, Sungkyunkwan University School of Medicine, Seoul, 03181 Republic of Korea; 6 Department of Otorhinolaryngology-Head and Neck Surgery, The Research Institute, Konkuk University School of Medicine, 120, Neungdong-ro, Seoul, 05029 Republic of Korea; 7 Faculty of Pharmacy, Ton Duc Thang University, Ho Chi Minh City, Vietnam

**Keywords:** Organoid, Salivary gland, Xerostomia, Stem cell therapy, Organoid transplantation

## Abstract

**Background:**

Xerostomia is a pathological condition characterized by decreased salivation due to salivary gland dysfunction and is frequently attributed to irreversible damage as a side effect of radiation therapy. Stem cell–derived organoid therapy has garnered attention as a promising avenue for resolving this issue. However, Matrigel, a hydrogel commonly used in organoid culture, is considered inappropriate for clinical use due to its undefined composition and immunogenicity. In this study, we aimed to develop a method for culturing collagen-based human salivary gland organoids (hSGOs) suitable for clinical applications and evaluated their therapeutic effectiveness.

**Methods:**

Human salivary gland stem cells were isolated from the salivary gland tissues and cultured in both Matrigel and collagen. We compared the gene and protein expression patterns of salivary gland–specific markers and measured amylase activity in the two types of hSGOs. To evaluate the therapeutic effects, we performed xenogeneic and allogeneic transplantation using human and mouse salivary gland organoids (hSGOs and mSGOs), respectively, in a mouse model of radiation-induced xerostomia.

**Results:**

hSGOs cultured in Matrigel exhibited self-renewal capacity and differentiated into acinar and ductal cell lineages. In collagen, they maintained a comparable self-renewal ability and more closely replicated the characteristics of salivary gland tissue following differentiation. Upon xenotransplantation of collagen-based hSGOs, we observed engraftment, which was verified by detecting human-specific nucleoli and E-cadherin expression. The expression of mucins, especially MUC5B, within the transplanted hSGOs suggested a potential improvement in the salivary composition. Moreover, the allograft procedure using mSGOs led to increased salivation, validating the efficacy of our approach.

**Conclusions:**

This study showed that collagen-based hSGOs can be used appropriately in clinical settings and demonstrated the effectiveness of an allograft procedure. Our research has laid the groundwork for the future application of collagen-based hSGOs in allogeneic clinical trials.

**Supplementary Information:**

The online version contains supplementary material available at 10.1186/s13287-024-03833-x.

## Background

Salivary glands, classified as exocrine glands, produce a complex fluid called saliva consisting of proteins, mucins, and enzymes that play a vital role in oral health maintenance [1, 2]. However, several factors, including Sjögren’s syndrome, aging, and the side effects of radiation therapy, can lead to decreased saliva production, resulting in a condition recognized as xerostomia [3, 4], which significantly impacts the quality of life, elevating the risks of dental caries, oral infections, and challenges in speaking and swallowing [5]. Xerostomia is one of the most severe and debilitating side effects in patients undergoing radiation therapy for head and neck cancer [6]. Xerostomia developing as a result of radiation exposure is often irreversible as radiation leads to the destruction of salivary gland stem cells, which are crucial for tissue regeneration [7–10].

Given the lack of a definitive solution for patients who have lost all their salivary gland stem cells, stem cell–based regenerative therapies offer a promising approach for restoring these cells and thereby repairing damaged salivary gland tissue [8]. Several types of adult stem cells have been utilized in the regeneration of damaged salivary glands, including bone marrow–derived stem cells (BMCs) [11], salivary gland–derived mesenchymal stem cells [12], and salivary gland epithelial cells [13].

In recent years, there has been growing interest in organoid-based regenerative medicine. Organoids are intricate three-dimensional (3D) structures that spontaneously form through self-assembly of stem cells, closely resembling the structural and functional characteristics of organs [14]. They have numerous applications, including extensive utilization in drug discovery and regenerative therapies [15–17]. Several studies have successfully developed salivary gland organoids that can self-renew and differentiate into various cell types within salivary glands. Pringle et al. (2016) established “Salispheres”, which enhance the regeneration of irradiated salivary glands and restoration of salivary secretion [18]. Sui et al. (2020) generated human salivary gland organoids and induced differentiation using mouse embryonic mesenchyme [19]. Yoon et al. (2022) established long-term cultures of mouse and human salivary gland organoids that exhibited cell type heterogeneity [20].

Despite extensive efforts, the clinical application of salivary gland organoids still faces significant challenges. Matrigel, a hydrogel commonly used for organoid culture, closely mimics the mechanical properties and composition of native extracellular matrix (ECM) [21]. However, because it is derived from mouse sarcoma cells, it is not clinically suitable owing to concerns regarding its undefined composition and immunogenicity [21–23]. Furthermore, the challenge of cultivating human salivary gland organoids on a large scale is a significant barrier to their therapeutic application. Hence, it is imperative to culture organoids under well-defined conditions to facilitate extensive cultivation [24].

In this study, we developed a method for the large-scale cultivation of Matrigel-based and collagen-based hSGOs. We verified the therapeutic potential of collagen-based hSGOs in damaged salivary glands using xenotransplantation in a xerostomia mouse model. However, allogeneic validation is necessary to confirm the clinical application of these organoids. To simulate a practical setting, we generated mSGOs and transplanted them into a radiation model, demonstrating their effectiveness in increasing salivary secretion. Taken together, our study provides a solid foundation for the clinical implementation of salivary gland organoids as a promising therapeutic option for salivary gland dysfunction.

## Materials and methods

### Isolation of stem cells and organoid culture in the human salivary gland

This study was approved by the Institutional Review Boards (IRB) of Konkuk University Hospital (IRB No. 202011062) and Kangbuk Samsung Hospital (IRB No. 2023-03-042-002), and all donors provided written informed consent. Normal submandibular and parotid gland tissues were obtained from non-damaged regions of patients with head and neck cancer. The tissues were minced and washed three times with PBS. Chopped tissues were enzymatically digested in HBSS/1% BSA buffer containing 0.63 mg/mL collagenase II (Invitrogen) and 0.5 mg/mL hyaluronidase (Sigma- Aldrich) by shaking (120 RPM) at 37 °C for 2 h.

For collagen culture, we used type 1 atelocollagen isolated from bovine hides (Cat.no# 5010; Advanced Biomatrix). Briefly, 5,000 cells in a 20-μL mixture of collagen and media (1:1 v/v) were seeded onto a 48-well plate and solidified to form a dome in 37 °C, 5% CO_2_ incubator. Collagen-based hSGOs culture medium contained AdDMEM/F12 (GIBCO) supplemented with GlutaMAX, HEPES, Penicillin-Streptomycin, B-27 (all Thermo Fisher Scientific), 1.25 mM N-Acetyl-L-cysteine (Sigma-Aldrich), 500 nM A83-01, 10 μM Y-27632 (all Tocris), 100 ng/mL FGF10, 100 ng/mL Noggin (all ORGANOIDSCIENCES), 10 mM Nicotinamide (Sigma-Aldrich) and 10% Wnt3a conditioned medium (ORGANOIDSCIENCES). The culture medium was changed every 2–3 days. For passage, organoids were collected at day 7 and incubated at 37 °C for 20 min with Liberase (Sigma-Aldrich) and for 5 min with 0.05% trypsin–EDTA for dissociation into single cells.

For Matrigel culture, 5,000 cells in a 20-μL mixture of Matrigel (Corning) and media (7:3 v/v) were seeded onto a 48-well plate and solidified to form a dome in 37 °C, 5% CO_2_ incubator. Matrigel-based hSGOs were cultured in expansion medium (EM, AdDMEM/F12 (GIBCO) supplemented with GlutaMAX, HEPES, Penicillin–Streptomycin (all Thermo Fisher Scientific), 1.25 mM N-Acetyl-L-cysteine (Sigma-Aldrich), 500 nM A83-01, 10 μM Y-27632 (all Tocris), 100 ng/mL FGF10, 100 ng/mL Noggin and 10% R-spondin 1 conditioned medium (all ORGANOIDSCIENCES)). The culture medium was changed every 2–3 days. For passage, organoids were collected on day 5 and incubated at 37 °C for 5 min with 0.05% trypsin–EDTA for dissociation into single cells.

### Isolation of stem cells and organoid culture in the mouse salivary gland

C57BL/6 N mice (Female, 7-weeks old) were obtained from JA BIO. The submandibular glands were weighed to adjust the enzyme volume, washed thrice with PBS, and minced. The remainder of the isolation procedure was the same as that used for human cell preparation. To establish the mSGOs, we adapted and modified the culture condition developed by Pringle et al. [18. 5,000 cells in a 20-μL mixture of Matrigel (Corning) and media (7:3 v/v) were seeded onto a 48-well plate and solidified. mSGOs were cultured in DMEM/F12 (GIBCO) supplemented with GlutaMAX, Penicillin–Streptomycin (all Thermo Fisher Scientific), 20 ng/mL EGF (Sigma-Aldrich), 20 ng/mL FGF2 (Sigma-Aldrich), N-2 (Invitrogen), 10 μg/mL Insulin (Sigma-Aldrich), 1 μM Dexamethasone and 50% WNT3a-R spondin1-Noggin–conditioned medium (WRN; ATCC). For passage, organoids were collected on day 5 and incubated at 37 °C for 5 min with 0.05% Trypsin–EDTA for dissociation into single cells.

### Differentiation of human and mouse salivary gland organoids

For salivary gland organoid differentiation, we coated 96-well plates with a 40-μL mixture of Matrigel: media (7:3 v/v) and incubated it for 20 min at 37 °C. Organoids obtained at days 5–6 were mixed with 50 μL of Matrigel: media (7:3 v/v) mixture and added into the coated wells and incubated for 20 min at 37 °C. For hSGOs, the differentiation medium (DM; AdDMEM/F12 (GIBCO) supplemented with GlutaMAX, HEPES, Penicillin–Streptomycin (all Thermo Fisher Scientific), 1.25 mM N-Acetyl-L-cysteine (Sigma-Aldrich), 5 nM Neuregulin (Peprotech), 10 μM Y-27632 (Tocris) and 100 ng/mL FGF10 (ORGANOIDSCIENCES) was added on top of the gel, and they are cultured for about additional 10 days. The culture medium was changed every 2–3 days. mSGOs undergo spontaneous differentiation up to 12 days; therefore, it was not necessary to use DM.

### Histological analysis

Tissue and organoids were fixed in 4% formaldehyde, processed, and embedded in paraffin wax. The 4-μm-thick sections were deparaffinized in xylene and rehydrated in a graded series of ethanol. Hematoxylin and eosin (H&E; Abcam) staining was performed according to standard protocols. To identify mucin production in tissue and organoids, we performed periodic acid–Schiff (PAS; Abcam) and Alcian blue (Abcam) staining. For PAS staining, sections were stained with PAS solution for 5 min at room temperature. After washing, sections were stained with Schiff’s solution for 15 min at room temperature. For Alcian blue staining, sections were stained with Acetic Acid solution for 3 min and Alcian Blue (pH 2.5) solution for 30 minutes at room temperature. Images were visualized using a TS2-LS microscope (Nikon).

### Immunofluorescence analysis

For immunofluorescence analysis, tissue and organoids sections were deparaffinized in xylene and rehydrated in a graded series of ethanol. The sections were heated in antigen retrieval buffer (10 mM Sodium citrate with 0.05% Tween 20, pH 6.0) for 20 min at 95 °C. After blocking with 5% normal horse serum (Vector Laboratories) for 1 h at room temperature, sections were incubated overnight at 4 °C with primary antibodies. The primary antibodies used for staining were: mouse anti-cytokeratin 19 (1:200, Biolegend); rabbit anti-cytokeratin 7 (Roche); mouse anti-α amylase (1:200, Santa cruz); rabbit anti-aquaporin 5 (1:200, Alomone labs); mouse anti-MUC5B (1:200, Abcam); rabbit anti-CD166 (1:200, Novus Biologicals); rabbit anti-Ki67 (1:300, Abcam); mouse anti-cytokeratin 17 (1:200, Santa cruz); mouse anti-cytokeratin 14 (1:200, Abcam); mouse anti-human nucleoli (1:100, Abcam); rabbit anti-E-cadherin (1:300, Abcam). The following day, sections were incubated with secondary antibodies for 1 h at room temperature. The secondary antibodies used were goat anti-mouse Alexa Fluor 488 (1:500) and goat anti-rabbit Alexa Fluor 594 (1:500) (all Invitrogen). Nuclei were stained with 1 μg/mL Hoechst 33342 (Sigma). Fluorescence staining was visualized using a TS2-LS microscope (Nikon).

### Quantitative real-time polymerase chain reaction (qRT-PCR)

Total RNA was extracted from hSGOs using TRIzol reagent (Invitrogen) following the manufacturer’s instructions. AccuPower RT PreMix (Bioneer) was used to synthesize cDNA from total RNA. qRT-PCR was performed with cDNA and SYBR^®^ Premix Ex Taq™ (Takara Bio) using a Thermal Cycler Dice Real-Time System III (Takara Bio). The comparative Ct method was used to normalize and calculate the relative mRNA levels of target genes.

### Amylase activity assay

hSGOs were harvested, and an amylase assay kit (Abcam) was used for this study following the manufacturer’s protocol. Briefly, organoids were broken down using amylase assay buffer. Cell lysates were collected by centrifugation and the supernatant was transferred to a new tube. Protein quantitation was performed using Bradford Protein Assay kit (Bio-Rad) and known amount of protein was mixed with the substrate solution provided in the assay kit. Absorbance was measured immediately at 405 nm every 3 min, for 60 min at 25 °C, with the plate protected from light over the entire duration.

### Calcium signaling assay

To investigate the intracellular calcium mobilization in hSGOs and mSGOs, we used Fluo-4 Calcium Imaging Kit (Thermo Fisher Scientific). On day 15, the organoids were exposed to Fluo-4 AM for 1 h at 37 °C, followed by 15 min of incubation at RT. After washing with PBS, the organoids were stimulated with 50 mM carbachol (Sigma). A Nikon Eclipse Ti2 microscope (Nikon) was used to observe calcium signaling.

### Animal experiments

All experiments have been reported in line with the ARRIVE guidelines 2.0. The CHA University Institutional Animal Care and Use Committee (IACUC, IRB no.210081) approved all animal experiments of this study. NSGA mice (Female, 8-weeks old, IACUC IRB no.220137) were purchased from JA BIO. C57BL/6-Tg (CAG-EGFP) mice (Female, 8- to 10-weeks old) were purchased from Japan SLC. All mice were housed in a constant temperature (approximately 23 °C) room on a 12-h light–dark cycle and fed *ad libitium*. Body weight was measured every 2 weeks throughout the experiment. All surgical procedures on mice were conducted under anesthesia using a combination of 100 mg/kg ketamine and 10 mg/kg xylazine administered intraperitoneally. For the method of mice euthanasia, we used carbon dioxide (CO_2_) inhalation.

### Establishment of a mouse model of xerostomia

The mice were irradiated using a medical linear accelerator (Clinac iX; Varian Medical Systems, Palo Alto, CA, USA) that generated 6 MV photons. The irradiation dose was 6 Gy/min. The salivary glands of mice were locally irradiated with a single X-ray dose of 7.5 Gy and 15 Gy for NSGA (*n* = 4) and C57BL/6 N (*n* = 13) mice, respectively, under anesthesia. This radiation dose induced salivary gland injury and decreased the saliva flow rate in the mice without compromising the general health of the animals, especially without causing esophagitis.

### Transplantation of the human and mouse salivary gland organoids

For hSGOs transplantation, 4 weeks post-radiation, a 5-mm incision was made in front of the neck of the irradiated NSGA mice (*n* = 4) under anesthesia to expose the submandibular gland. Human parotid gland organoids were labeled with Cytopainter (Cat.no# ab176735; Abcam) according to the manufacturer’s instructions. Whole organoids (about 4,000 organoids/10^5^ cells) were locally injected to both glands. The wounds were then closed with sutures. For mSGOs transplantation, C57BL/6-Tg (CAG-EGFP)-mSGOs were prepared as a single-cell suspension (1x10^4^ or 1x10^5^ cells/20 μL) and directly transplanted into both salivary glands in irradiated C57BL/6 N mice (*n* = 13) using a 29-gauge Hamilton syringe. Control group (Age-matched mice that did not receive irradiation and transplantation, *n* = 3) underwent surgery only and vehicle group (Irradiated mice that did not receive transplantation, *n* = 4) was locally injected with PBS. Vehicle and transplantation groups were randomized, ensuring there was no statistical significance based on body weight and salivation. No animals were excluded from the experiment.

### Measurement of saliva secretion in the in vivo model

To visualize the saliva secretion of recipient mice, each mouse was subcutaneously injected with pilocarpine (1 mg/kg) with fluorescein paper (Haag–Streit Diagnostics) being placed on the tongue of the mouse for 10 min under anesthesia. Saliva secretion was measured a day before exposure to radiation and every 4 weeks thereafter, with those who measure saliva flow being unaware of the allocation groups throughout the experiment.

### Single-cell RNA sequencing

Human submandibular gland tissues and organoids were dissociated into single cells. They were resuspended in PBS containing 1% bovine serum albumin. Cells were counted automatically using Countess II (Thermo Fisher, Waltham, MA) to determine their concentration. Single-cell RNA-sequencing libraries were the prepared using the Chromium Next GEM Single Cell 3’ reagent kit v3.1 (10X Genomics, Pleasanton, CA) in accordance with the manufacturer’s protocol. Briefly, the cells were diluted in the Chromium Next GEM Chip G to yield a recovery of approximately 5,000 single cells. Following library preparation, the libraries were sequenced in multiplex using a Novaseq 6000 sequencer (Illumina, San Diego, CA) to produce, on average, a minimum of 30,000 reads per cell.

### Analysis of single-cell RNA sequencing data

Sequencing reads were processed using Cell Ranger version 3.0.1 (10X Genomics, CA) using the human reference transcriptome GRch38 from Ensembl. From the gene expression matrix, downstream analysis was performed using R version 3.6.2. Quality control, filtering, data clustering, visualization, and differential expression analysis were performed using the WinSeurat program (Ebiogen Inc., Korea) based on the Seurat version 3.1.2 R package (Butler et al., 2018) with some custom modifications to the standard pipeline. For each dataset, the genes expressed in cells with < 500 UMIs and < 300 genes were removed from the gene expression matrix. In addition, we removed any single cells with > 10% UMIs mapped to mitochondrial genes. After scTransform-normalizing the data, the expression of each gene was scaled by regressing the number of UMI and the percentage of mitochondrial genes expressed in each cell. We performed PCA on the gene expression matrix and used the first 10–30 principal components for clustering and visualization. Clustering was performed with a resolution of 0.6–0.8 and visualization was done using uniform manifold approximation and projection (UMAP). Cell types were annotated using the following references [20, 25–33].

### Gene expression microarray

Undifferentiated and differentiated hSGOs were used for analysis. The data were summarized and normalized using the robust multi-average (RMA) method implemented in Affymetrix Power Tools (APT). We exported the result with gene-level RMA analysis and performed differentially expressed gene (DEG) analysis. The statistical significance of the expression data was determined using fold change. For each DEG set, hierarchical cluster analysis was performed using complete linkage and Euclidean distance as measures of similarity. Gene-Enrichment and Functional Annotation analyses for the significant probe list were performed using Gene Ontology (http://geneontology.org) and KEGG (http://kegg.jp). All data analyses and visualization of DEGs were conducted using R 3.3.2 (www.r-project.org).

### Statistical analysis

Experiments were repeated three times, and all data was reported as the mean ± SD. GraphPad Prism software, version 5.0 and 7.0, were used to calculate statistical significance using one-way analysis of variance (ANOVA) with a post hoc Tukey test or using two-way ANOVA with Tukey’s multiple comparison test (GraphPad Prism, CA, USA). The threshold for statistical significance was *p* < 0.05. Saliva secretion by factor (group and time) was analyzed using two-way ANOVA with Tukey’s multiple comparison test in the mixed effect model. Tissue weight was analyzed using the Mann–Whitney test between the control and IR groups and one-way ANOVA with Dunn’s multiple comparisons test for IR vs. two IR-TP groups.

## Results

### Establishment of hSGOs in Matrigel-based culture and identification of cellular origin

**Fig. 1:**
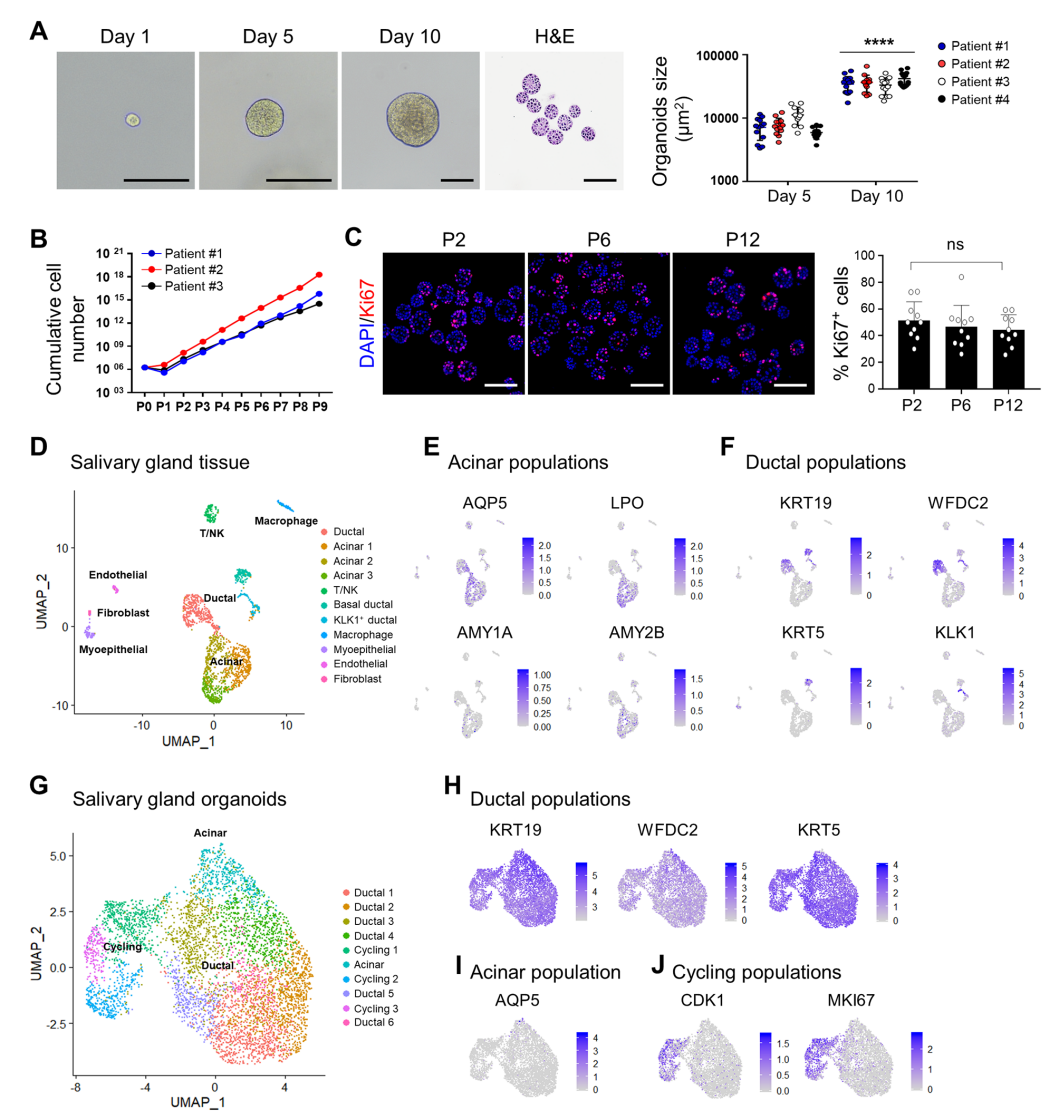
Generation of hSGOs in Matrigel-based culture and scRNA-seq analysis. (**A**) Microscopic image of the growth of hSGOs cultured in optimized medium and stained with Hematoxylin and Eosin (H&E) on day 5 of passage 5 (Left). Scale bar, 100 μm. Measurement of size of organoids (μm^2^) on day 5 and 10 (Right). *n* = 4, biologically independent samples. **** *p* < 0.0001 (**B**) Cumulative cell number of passage 0 to 9. *n* = 3, biologically independent samples. (**C**) Immunofluorescence staining of proliferative cell marker Ki67 in hSGOs on day 5 of passages 2, 6, and 12 (Left), percentage of Ki67-positive cells in hSGOs (Right). Scale bar, 100 μm. (**D**) Uniform manifold approximation and projection (UMAP) analysis of human submandibular gland tissue (*n* = 1,965 cells). (**E**) The expression of acinar cell markers (*AQP5*, *LPO*, *AMY1A*, and *AMY2B*) within the acinar cell clusters. (**F**) The expression of ductal cell markers (*KRT19*, *WFDC2*, *KRT5*, and *KLK1*) within the ductal cell clusters. (**G**) UMAP analysis of hSGOs at day 5 of passage 5 (*n* = 5,220 cells). (**H**) The expression of ductal cell markers (*KRT19*, *WFDC2*, and *KRT5*) within the ductal-like cell clusters, (**I**) Acinar cell marker (*AQP5*) within the acinar-like cell cluster, and (**J**) Cycling cell markers (*CDK1* and *MKI67*) within the cycling cell clusters

Prior to optimizing the culture conditions in collagen matrix, we first established these conditions using Matrigel, which has been widely used for organoid growth. Although many studies have reported the culture of human salivary gland organoids, we developed a new culture condition that further prolongs their proliferation and maintenance period. We isolated stem cells from salivary gland tissue, embedded in Matrigel, and cultured in an expansion medium (EM). By day 5, spherical hSGOs were observed, displaying a continuous increase in size over time (Fig. 1A). To sustain their growth, organoids were passaged every 5 days, resulting in rapid proliferation, with an average passage ratio of 1:30 (Fig. 1B). Importantly, the proliferative cell marker Ki67 was predominantly enriched in the outer layer of hSGOs and was consistently expressed throughout multiple passages, indicating robust self-renewal capacity (Fig. 1C).

To investigate the cellular diversity of salivary gland tissue and organoids, we performed single-cell RNA sequencing analysis. In the analysis of the tissue sample, we observed 11 distinct clusters (Fig. 1D). Among these, three clusters exhibited high expression levels of the *AQP5*, *LPO*, and *AMY* family genes, which are characteristic of acinar cells (Fig. 1E). Three other clusters were identified as ductal cells based on the expression of *KRT19*, *WFDC2*, *KRT5*, and *KLK1* (Fig. 1F). The remaining clusters were identified as myoepithelial cells (*ACTA2* and *MYH11*), endothelial cells (*VWF* and *PLVAP*), fibroblasts (*DCN* and *COL1A1*), T/NK cells (*CD3D* and *NKG7*), and macrophages (*AIF1* and *APOE*) (Fig. S1).

In the analysis of day 5 hSGOs cultured in EM, we observed that the majority of clusters exhibited enrichment in ductal cell markers such as *KRT19*, *WFDC2*, and *KRT5* (Fig. 1G and H). Only one cluster exhibited characteristics of acinar cells expressing *AQP5*, albeit in small populations (Fig. 1I). The remaining clusters were associated with actively dividing cell populations expressing *MKI67* and *CDK1* (Fig. 1J). Apart from epithelial cells, no other cellular types were detected in hSGOs, including immune cells, vascular cells, or fibroblasts. Overall, our findings indicated that under expansion conditions, the majority of hSGOs exhibited ductal cell–like features rather than those of acinar cells.

### Multilineage differentiation and functional replication of hSGOs in Matrigel-based culture

**Fig. 2:**
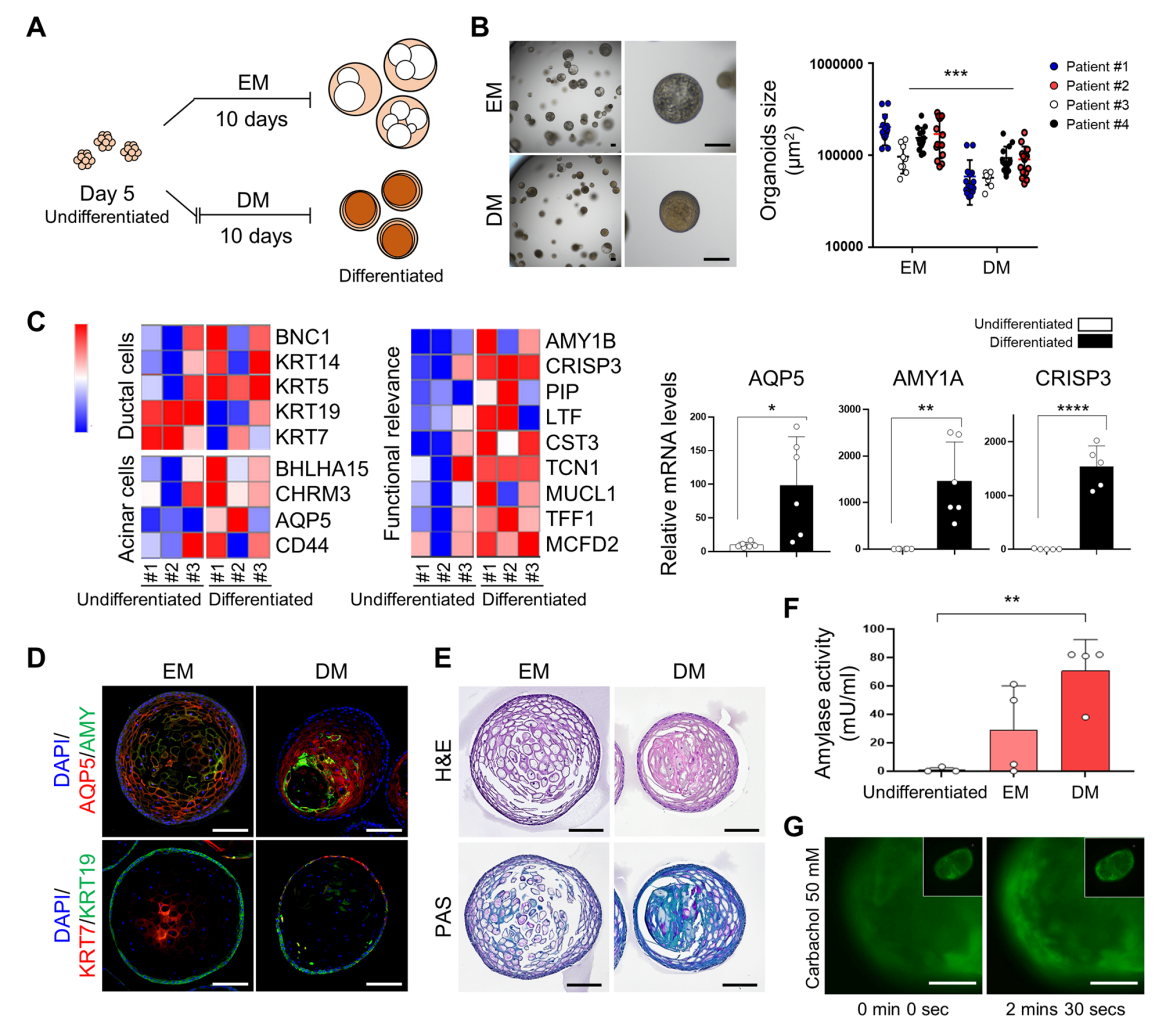
Differentiation and functional replication of hSGOs cultured in Matrigel. (**A**) Scheme of differentiation of hSGOs. On day 5, the expansion medium (EM) was replaced with differentiation media (DM), and the organoids were cultivated for an additional 10 days. (**B**) Microscopic images of organoids cultured in EM and DM (Left). Size of hSGOs in the EM and DM (Right). Scale bar, 100 μm. *n* = 4, biologically independent samples. *** *p* < 0.001. (**C**) Gene expression of acinar cell, ductal cell, and functional relevance markers of hSGOs on days 5 in EM (Undifferentiated) and day 15 in DM (Differentiated) (Left). Relative gene expression levels of *AQP5*, *AMY1A*, and *CRISP3* (Right). *n* = 5 or 6, biologically independent samples. * *p* < 0.05, ** *p* < 0.01, **** *p* < 0.0001. (**D**) Immunofluorescence staining of acinar cell markers (AQP5 and AMY) and ductal cell markers (KRT19 and KRT7) in hSGOs on day 15 in EM and DM. Scale bar, 100 μm. (**E**) H&E and periodic acid-Schiff (PAS) staining of hSGOs on day 15 in EM and DM. Scale bar, 100 μm. (**F**) Amylase activity of hSGOs cultured on days 5 (Undifferentiated) and days 15 (EM and DM). *n* = 4, biologically independent samples. ** *p* < 0.01. (**G**) Calcium influx measurement using Fluo-4 AM in hSGOs on day 15 in DM following stimulation with 50 mM carbachol. Scale bar, 100 μm

Under expansion conditions, hSGOs predominantly exhibited ductal cell–like features and had a limited resemblance to acinar cells. No spontaneous differentiation was observed in the EM. Therefore, to assess the potential of hSGOs to differentiate into multiple lineages of the salivary gland, we developed a differentiation medium (DM) by excluding conditioned medium, A83-01, and noggin from the EM. On day 5 of culture, we changed the medium from EM to DM and cultured the organoids for an additional 10 days (Fig. 2A). The results showed clear morphological differences in the organoids after they were transferred from EM to DM. Organoids cultured in EM exhibited a gradual loss of cellular components and an increase in size, whereas those cultured in DM showed an increase in cellular content (Fig. 2B). Remarkably, the gene expression profile of the DM-cultured organoids showed a distinct pattern compared to that of the undifferentiated organoids. Signature genes associated with ductal cells (*KRT5* and *KRT14*) and acinar cells (*BHLHA15*, *CHRM3*, and *AQP5*) were enriched in DM-cultured organoids. Additionally, the expression of genes functionally relevant to the salivary gland, such as *AMY1A*, *AMY1B*, *LTF*, *TCN1*, and *MCFD2* was upregulated (Fig. 2C).

Subsequently, we analyzed the expression of cell type–specific marker proteins in the organoids. In the DM-cultured organoids, AQP5 and AMY were predominantly expressed in the inner layer, whereas KRT7 and KRT19 were in the outer layer (Fig. 2D). Furthermore, neutral mucin, a characteristic secretion of mucous acinar cells, was enriched in DM-cultured organoids (Fig. 2E). Overall, these findings indicate that in the undifferentiated state, organoids possess the characteristics of ductal cells, and upon differentiation, they acquire the features of acinar cells.

Assessing the extent to which salivary gland organoids can replicate the function of salivary glands is important. Therefore, we performed functional assays. The amylase activity was measured, and the analyses revealed that organoids cultured in DM showed significantly elevated enzymatic activity compared to those cultured in EM (Fig. 2F). Next, because salivary gland function is regulated by neurotransmitters from the parasympathetic nervous system, we evaluated the calcium response, which is essential for salivation. Upon stimulation with the cholinergic agonist carbachol, DM-cultured organoids exhibited calcium accumulation (Fig. 2G). Taken together, our findings suggest that hSGOs possess the capability to differentiate into various cell types, including acinar and ductal cells, while mimicking the functional characteristics of salivary glands.

### Establishment of hSGOs in collagen-based optimized culture conditions for clinical application

**Fig. 3:**
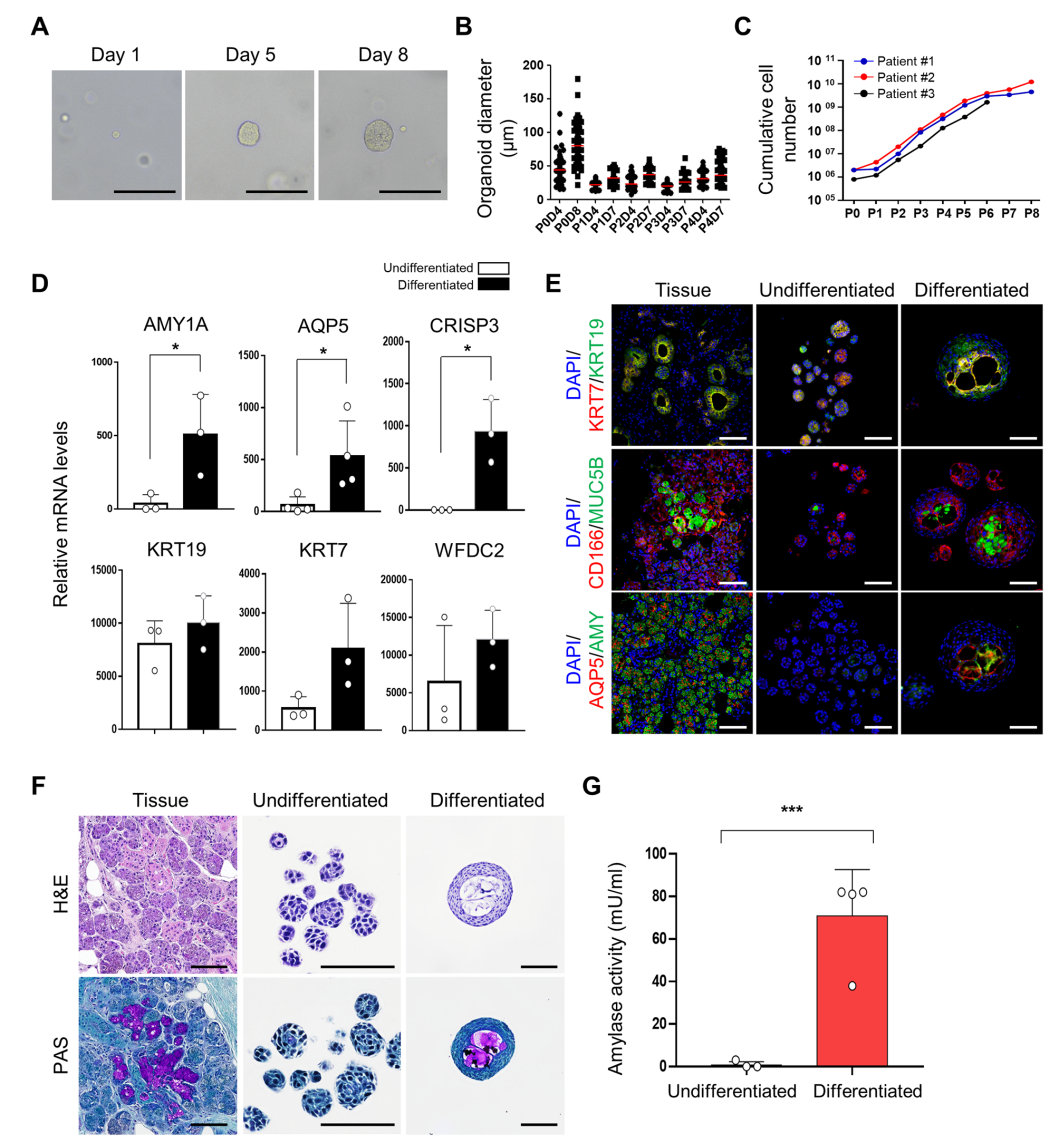
Generation and characterization of collagen-based hSGOs. (**A**) Microscopic image of the growth of hSGOs cultured in a type I collagen matrix. Scale bar, 100 μm. (**B**) Organoid diameters at days 4 and 7 of passages 1 to 4. (**C**) Cumulative cell number of passage 1 to 8. *n* = 3, biologically independent samples. (**D**) Gene expression of the acinar cell markers (*AMY1A*, *AQP5*, and *CRISP3*) and ductal cell markers (*KRT19*, *KRT7*, and WFDC2) of undifferentiated and differentiated organoids. *n* = 3 or 4, biologically independent samples. * *p* < 0.05. (**E**) Immunofluorescence staining for acinar cell markers (AQP5, AMY, CD166, and MUC5B) and ductal cell markers (KRT7 and KRT19) in undifferentiated and differentiated hSGOs and tissue. Scale bar, 100 μm. (**F**) H&E and PAS staining in undifferentiated and differentiated hSGOs and tissue. Scale bar, 100 μm. (**G**) Measurement of amylase activity of undifferentiated and differentiated hSGOs. *n* = 4, biologically independent samples. *** *p* < 0.001

Matrigel is derived from mouse sarcoma cells and its composition is not yet clearly known. Therefore, it is considered unsuitable for clinical use because of safety concerns. Consequently, we transitioned from the use of Matrigel to a collagen-based approach, which is considered more appropriate for clinical applications. Primary human salivary gland stem cells were cultured within a three-dimensional collagen matrix, and the media composition was optimized from that used in Matrigel-based culture. After 7 days, collagen-based hSGOs exhibited spheroid morphology and sustained growth through subsequent passages (Fig. 3A and B). The maintenance period of the collagen-based hSGOs was shorter than that of the Matrigel-based hSGOs. However, within a few passages, the collagen-based hSGOs exhibited rapid proliferation, leading to the generation of a substantial number of cells for transplantation (Fig. 3C).

Upon the induction of differentiation, collagen-based hSGOs exhibited noticeable morphological changes, characterized by the progressive development of internal cavities, distinct from Matrigel-based hSGOs. They displayed upregulated expression of salivary gland markers associated with acinar cells (*AMY1A*, *AQP5*, and *CRISP3*) and ductal cells (*KRT19*, *KRT7*, and *WFDC2*) (Fig. 3D). Importantly, distinct protein expression patterns were observed compared to Matrigel-based hSGOs. The differentiated collagen-based hSGO exhibited polarized expression of the ductal cell markers KRT7 and KRT19, along with the development of a lumen (Fig. 3E). The presence of a lumen and polarized marker expression patterns are characteristic of duct-like structures [34]. Equally significant, the expression of the acinar cell markers CD166 and AQP5, along with the secretion markers MUC5B and AMY, indicated that differentiated organoids contain functional acinar cells. (Fig. 3E). Additionally, neutral mucin was enriched in the collagen-based differentiated hSGOs (Fig. 3F).

Next, we measured amylase activity to validate the functionality of collagen-based hSGOs. The results revealed significantly higher levels of functional amylase expression in differentiated collagen-based hSGOs than in undifferentiated hSGOs (Fig. 3G). In summary, we confirmed that collagen-based hSGOs cultured in the optimized medium demonstrated sufficient self-renewal and differentiation capabilities, comparable to those of Matrigel-based hSGOs.

### Engraftment, growth, and polarization of collagen-based hSGOs in a xerostomia mouse model

**Fig. 4:**
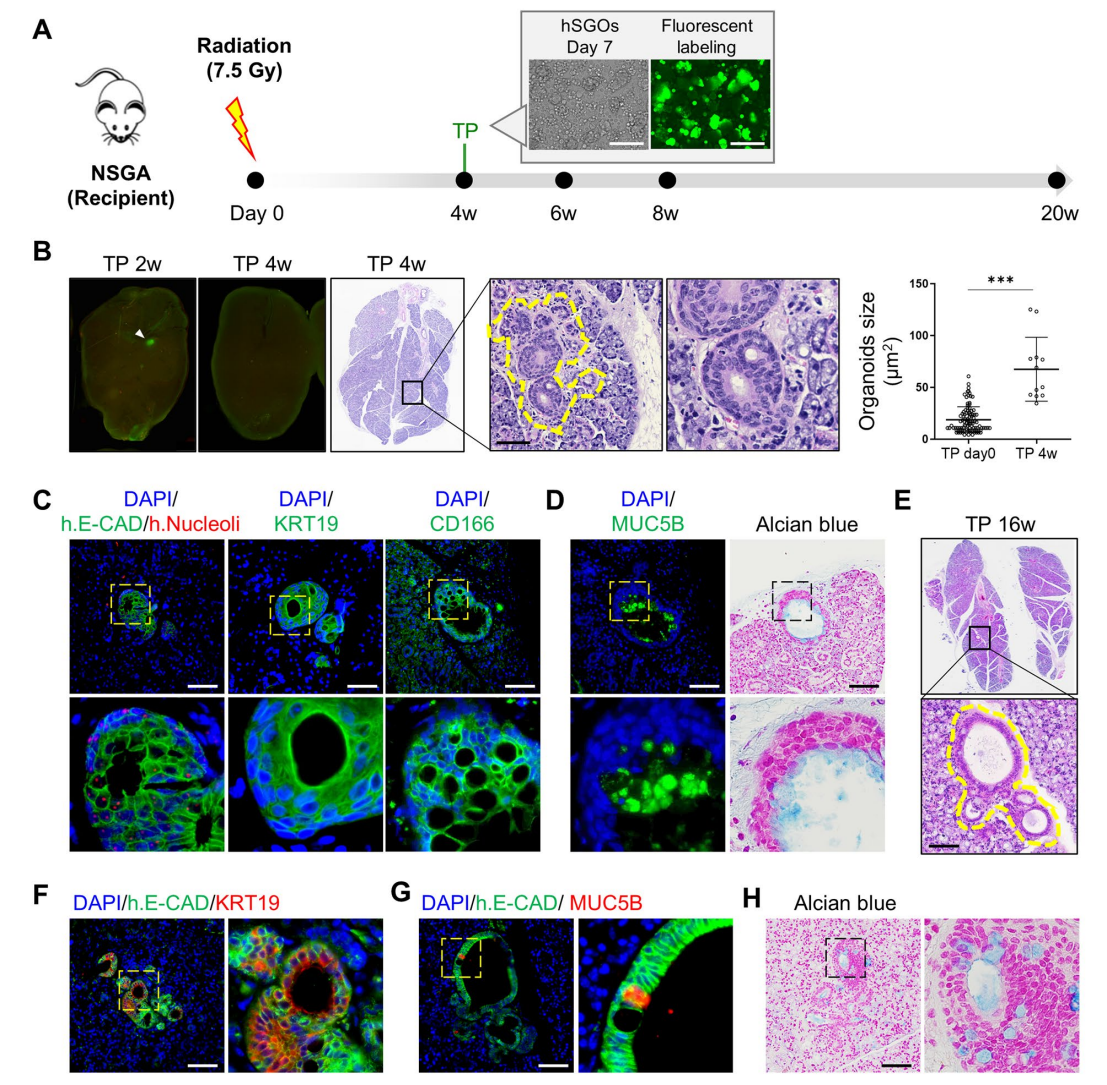
Transplantation of collagen-based hSGOs in a xerostomia mouse model. (**A**) Strategy for transplantation of hSGOs to a radiation-induced xerostomia mouse model. Scale bar, 250 μm. (**B**) Engrafted region of parotid gland organoids labeled with Cytopainter at 2 and 4 weeks (Left, white arrowhead indicate an engrafted region). Historical analysis revealed large nuclei-consisting organoids in the recipient’s submandibular gland at 4 weeks post-transplantation (Right, yellow dashed line). Scale bar, 100 μm. *** *p* < 0.001. (**C**) Human E-cadherin, human nucleoli, KRT19, CD166 were detected in the engrafted organoid of the recipient’s submandibular gland by immunofluorescence staining. Scale bar, 100 μm. (**D**) Immunofluorescence staining with MUC5B and Alcian blue staining for mucin secretion. Scale bar, 100 μm. (**E**) Historical analysis of integrated organoids on 16 weeks post-transplantation (Yellow dashed line). Scale bar, 100 μm. (**F**) Expression of human E-cadherin, KRT19, and (**G**) MUC5B in engrafted hSGOs at 16 weeks post-transplantation. Scale bar, 100 μm. (**H**) Alcian blue staining for mucin secretion in engrafted hSGOs at 16 weeks post-transplantation. Scale bar, 100 μm

To investigate whether the collagen-based hSGOs could integrate into the damaged salivary gland tissue and play a functional role, we performed xenotransplantation. We established the immunodeficient NSGA xerostomia model by subjecting the neck area of NSGA mice to irradiation at 7.5 Gy. After 4 weeks, the mice received direct transplantation of whole parotid gland organoids cultured in collagen into the submandibular glands. To validate the engraftment of hSGOs, the in vivo visualization of human organoids was facilitated by labeling them with Cytopainter, a fluorescent dye, for localization (Fig. 4A). At 2 weeks post-transplantation, Cytopainter-stained organoids were observed. However, according to the manufacturer, Cytopainter can only track up to nine generations of cells. Consequently, fluorescence was not observed at 4 weeks post-transplantation (Fig. 4B, Left). Instead, we tracked the engrafted organoids using a human-specific E-cadherin or human nucleolar antibody.

At 4 weeks post-transplantation, we analyzed H&E-stained salivary gland tissue sections from recipient mice and identified the engraftment of hSGOs at the central region of the salivary gland (Fig. 4B, Right). We observed the expression of human nucleoli and human E-cadherin in the engrafted organoids. They expressed the ductal cell marker KRT19 and acinar cell marker CD166 [35] (Fig. 4C). These findings suggest that engrafted hSGOs possess the capability to differentiate into multiple lineages. Subsequently, we investigated whether the transplanted hSGOs played a functional role. Specifically, we selected MUC5B as a functional marker essential for the lubrication of the oral mucosa. Indeed, we observed both mucin secretion and the expression of MUC5B in the transplanted hSGOs, implying an improvement in salivary composition (Fig. 4D).

At 16 weeks post-transplantation, we observed the expansion of organoids and formation of lumens (Fig. 4E). Human E-cadherin was constitutively expressed, along with MUC5B and KRT19 (Fig. 4F and G), and mucin secretion was detected (Fig. 4H). Overall, our results demonstrate the successful engraftment of collagen-hSGOs in a xerostomia mouse model, with differentiation into multiple lineages of salivary glands.

### Establishment and structure recapitulation of mouse salivary gland organoids (mSGOs)

We demonstrated engraftment and proliferation of hSGOs in an immunodeficient mouse model of xerostomia. However, this model does not replicate allotransplantation, which closely resembles clinical transplantation. To address this, we established mSGOs and performed allotransplantation in a xerostomia mouse model. The growth rate of mSGOs decreased when cultured in the same niches as the collagen-based hSGO cultures (data not shown). Therefore, we cultured mSGOs in Matrigel and modified the medium to suit mSGOs cultures.

**Fig. 5:**
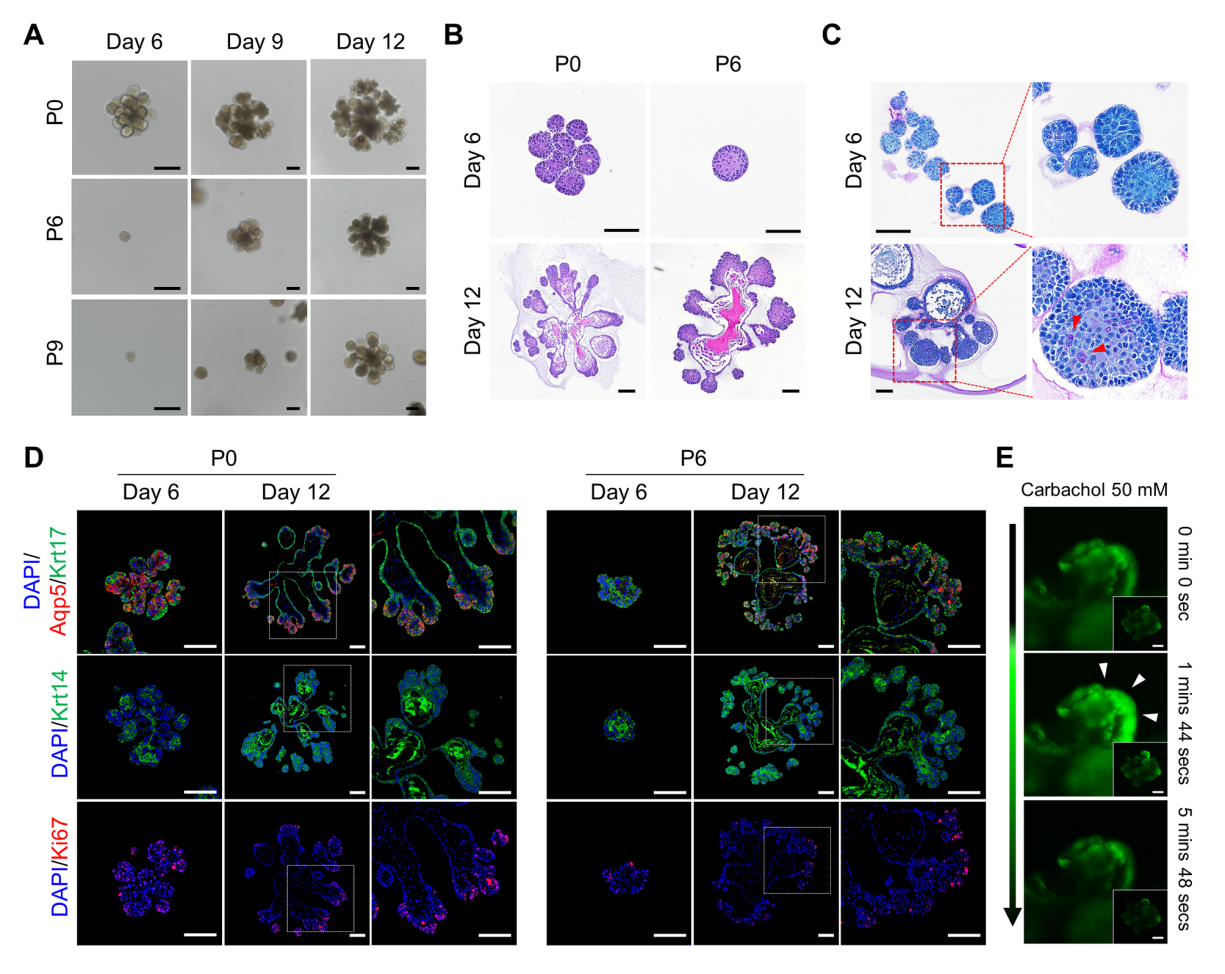
Generation and structure recapitulation of mSGOs. (**A**) Microscopic image of the growth of mSGOs on days 6, 9, and 12 of passage 0, 6, and 9. Scare bar, 100 μm. (**B**) H&E staining of mSGOs on days 6 and 12 of passage 0 and 6 for identifying the acini and duct structure. Scale bar, 100 μm. (**C**) PAS staining of mSGOs on days 6 and 12 of passage 6 for identifying the secretion of mucin (Red arrowhead). Scale bar, 100 μm. (**D**) Immunofluorescence staining for acinar cell marker (Aqp5), ductal cell markers (Krt17 and Krt14), and proliferative cell marker (Ki67) in mSGOs on days 6 and 12 of passage 0 and 6. Scare bar, 100 μm. (**E**) Calcium signaling assay of mSGOs on day 12. Intracellular calcium mobilization was observed after stimulation with 50 mM carbachol. Scale bar, 100 μm

mSGOs were generated by day 6, with mSGOs at P0 exhibiting budded and branched morphologies. At day 6 of P6 and P9, mSGOs exhibited spheroid shapes. However, both mSGOs exhibited spontaneous differentiation with morphological changes for up to day 12 (Fig. 5A). Therefore, the structural features of the salivary glands were recapitulated in the mSGOs and maintained even after repeated passages. Morphological similarities, including branches and buds indicative of salivary gland properties, were observed in the mSGOs (Fig. 5B). Mucin secretion indicated the ability of the mSGOs to produce mucin (Fig. 5C). Notably, mSGOs exhibited region-specific expression of salivary gland markers, with Aqp5 expressed in every bud region, Krt17 in the edge region, and Krt14 in the central region. Ki67, which indicates constant proliferation, was also expressed in the bud region (Fig. 5D). Furthermore, the mSGOs exhibited functional capabilities, including intracellular calcium mobilization, upon carbachol treatment (Fig. 5E). Collectively, our findings demonstrated that mSGOs replicated the structure and function of mouse salivary glands.

### mSGOs increase salivary secretion via the regeneration of damaged acini

**Fig. 6:**
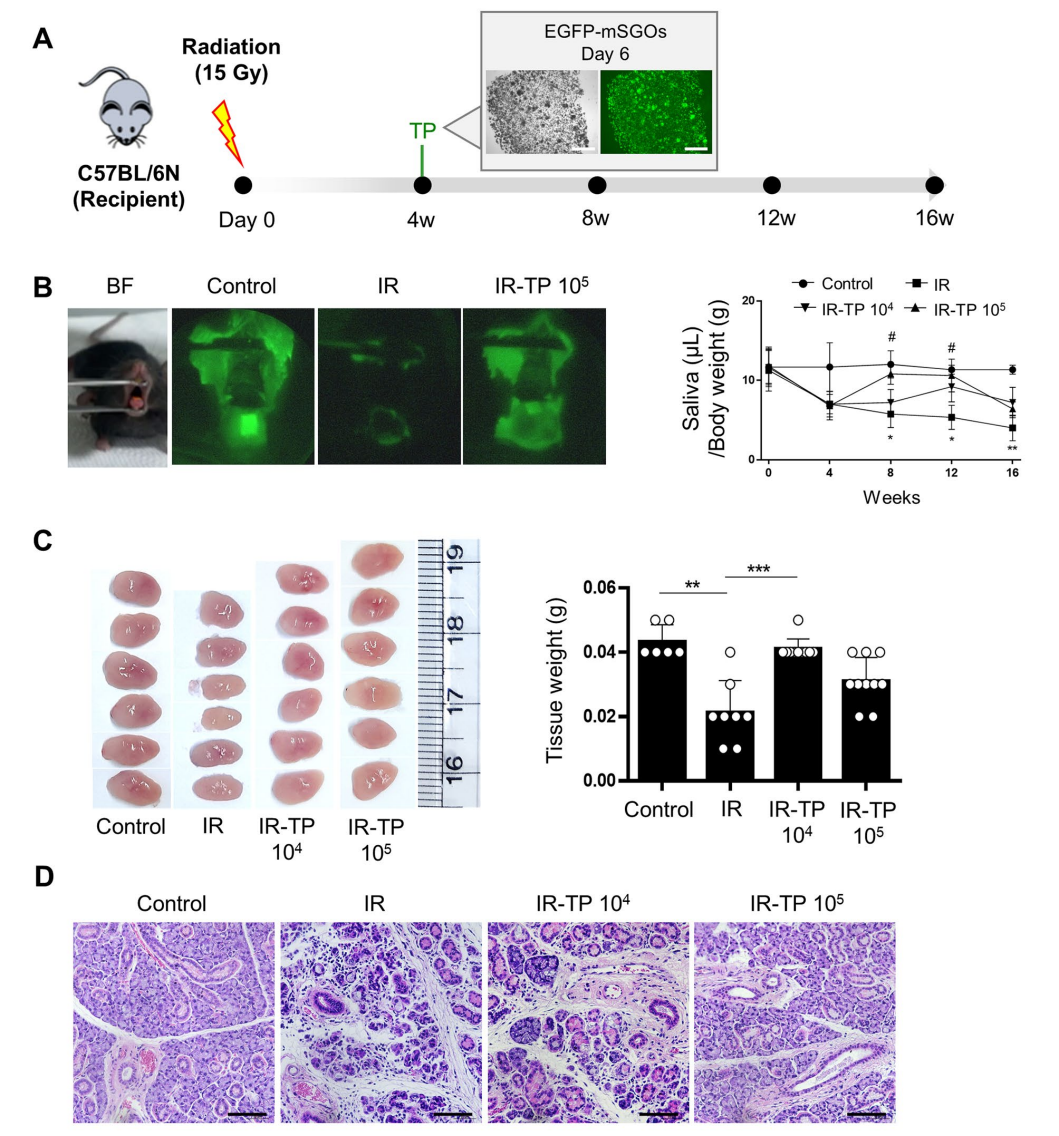
Transplantation of mSGOs in a xerostomia mouse model. (**A**) Strategy for transplantation of EGFP-mSGOs cells in radiation-induced xerostomia model. (**B**) Visualization using fluorescein paper for saliva secretion at 8 weeks post-radiation. Fluorescence intensity was measured by opening the mouth using tweezers (Left). Graph shows the measurement of saliva secretion by time (Right). *n* ≥ 3 mice per group. Mean ± SD. *, Control vs. IR, *p* < 0.05; #, IR vs. IR-TP 10^5^, *p* < 0.05. IR, irradiation; IR-TP 10^4^ and 10^5^, irradiation-transplantation and doses (cell number). (**C**) Representative gross image of salivary glands at the age-matched controls and at 16 weeks post-radiation (Left). Comparison of salivary gland tissue weight (Right). *n* ≥ 3 mice per group. Bar, mean ± SD. **, Control vs. IR, *p* < 0.01; ***, IR vs. IR-TP 10^4^, *p* < 0.001. (**D**) A comparison of histological analysis using H&E staining. Salivary gland tissues from the age-matched controls and at 16 weeks post-radiation. Scale bar, 50 μm

We investigated the regenerative capacity of mSGOs for salivary gland damage in a mouse model of radiation-induced xerostomia. C57BL/6 N mice received local irradiation at 15 Gy in the neck area. 4 weeks post-radiation, 10^4^ or 10^5^ cells of mSGOs were transplanted into the recipients (Fig. 6A). 8 weeks post-radiation, we visualized saliva secretion using fluorescein paper. Fluorescence intensity was measured by opening the mouth using tweezers. Pilocarpine, a parasympathomimetic drug that stimulates salivation by binding to the M3 muscarinic receptor in the salivary glands, was administered along with a fluorescein paper placed in the mouth for 10 min. Salivary secretion was significantly reduced by radiation in the irradiation-only group (IR), whereas the non-IR (Control) and 10^5^ organoid cell transplantation groups (IR-TP) exhibited increased salivary secretion, as indicated by fluorescein intensity (Fig. 6B, Left). Additionally, salivary secretion, which was measured every 4 weeks throughout the experiment, increased in the IR-TP 10^5^ group from 4 to 8 weeks post-transplantation (Fig. 6B, Right). Mice that underwent irradiation experienced tissue weight loss in the salivary glands. The IR group did not show any improvement of this weight loss, whereas the organoid cell transplantation groups (IR-TP 10^4^ and IR-TP 10^5^) showed recovery of tissue weight (Fig. 6C). There were no significant differences in body weight among the groups (data not shown). Furthermore, organoid transplantation facilitated the regeneration of damaged acini caused by radiation (Fig. 6D). Transplanted organoids derived from EGFP mice were detected in only one subject in the IR-TP 10^4^ group (*n* = 5) and four subjects in the IR-TP 10^5^ group (*n* = 5) after 12 weeks of radiation (data not shown). These results suggest that mSGOs have therapeutic potential for addressing acinar damage in a radiation-induced xerostomia model, with effects lasting for approximately 8 weeks.

## Discussion

Xerostomia is characterized by a significant reduction in saliva production, and organoid transplantation holds promise as a therapeutic option for a fundamental solution. The use of organoids derived from adult stem cells for tissue regeneration has attracted considerable attention in recent years. In this study, we generated human salivary gland organoids in Matrigel and collagen using novel culture conditions for large-scale cultivation. Furthermore, we simulated a clinical setting by performing xenogeneic and allogeneic transplantation of hSGOs and mSGOs, providing insights into the potential clinical applications of salivary gland organoids.

Previous studies have encountered challenges in promoting the proliferation of human salivary gland organoids [18, 20]. To address this issue, we conducted media screening and identified optimal growth conditions. Fibroblast growth factor 10 (FGF10) promotes salivary gland morphogenesis in mice [19]. The Rho kinase (ROCK) inhibitor Y27632 enhances pro-acinar cell proliferation and reduces apoptosis in vitro [36]. Further, noggin, R-spondin, Wnt and A83-01 have been shown to maintain stem cell characteristics in organoids [18, 37]. The inclusion of these factors facilitated the rapid expansion of the organoids, with a maximum passage ratio of 1:30. Our findings suggest that the organoid culture medium developed in this study promotes more rapid organoid growth than previous conditions with similar culture durations.

By conducting scRNA-seq analysis, we identified the specific population of salivary gland tissue from which the salivary gland stem cells were derived. The organoids exhibited elevated expression of genes associated with ductal cells in salivary gland tissue. Numerous studies have provided evidence that progenitor cells responsible for salivary gland regeneration are located within the ductal regions [38]. Our findings align with these hypotheses as the majority of undifferentiated cells in organoids displayed ductal characteristics.

Stem cells within the salivary glands possess a unique capacity to differentiate into various cell types. Our objective was to explore whether stem cells could differentiate into acinar, myoepithelial, and ductal cells. Through specific modifications to culture conditions, we successfully induced the differentiation of stem cells into both ductal and acinar cells.

Specifically, we achieved the differentiation hSGOs into functional acinar cells capable of producing amylase. Moreover, hSGOs displayed responsiveness to neurotransmitters and exhibited calcium mobilization, which is a critical process involved in fluid secretion within the salivary glands [39]. As the organoids did not replicate the complex branching and budding structures of the salivary gland tissue, we developed an additional culture condition to address this limitation (data not shown). However, the level of amylase production under these conditions was too low to confirm the organoid function; therefore, we opted for the current method. Overall, our data demonstrated the potential of hSGOs to differentiate into multiple cell types.

To develop a more clinically suitable approach, we replaced the Matrigel with collagen, a more defined hydrogel, and modified the culture medium accordingly. Collagen-cultured organoids demonstrate rapid growth and long-term maintenance. Importantly, these organoids exhibited the ability to differentiate into multiple lineages, similar to organoids cultured in Matrigel, indicating that collagen-based organoids share comparable characteristics with their Matrigel counterparts.

Following transplantation of collagen-based hSGOs into a mouse model of xerostomia, we observed successful integration of the organoids into host tissues, leading to the differentiation of ductal cells and MUC5B-producing acinar cells. MUC5B plays a crucial role in lubricating the oral mucosa and its levels are significantly diminished in patients with xerostomia [40]. The therapeutic effectiveness of xerostomia might depend not only on the quantity of saliva but also on its composition. Subjective dryness, which can affect the mucosal wettability of the oral surface, may be linked to changes in salivary composition [41]. Therefore, we suggest that hSGOs expressing MUC5B may improve salivary composition in xerostomia models.

This study had certain limitations. In the xenotransplantation experiment, hSGOs were engrafted in a small area compared to the entire salivary gland. The limited extent of engraftment could potentially result in a lack of improvement in salivary flow. Engraftment and survival of transplanted stem cells are crucial for enhancing the function of damaged organs [42, 43]. Addressing the low engraftment rate is another challenge that must be overcome through further investigation. Overall, we observed that the transplanted hSGOs not only successfully integrated into the mouse salivary gland tissue, but also utilized the host’s metabolism for growth and differentiation. Although we did not observe an increase in salivary secretion, our findings emphasize the potential of organoids to alleviate dryness and enhance lubrication through the production of MUC5B. Furthermore, considering that this was a xenotransplantation, we anticipated an increase in salivary secretion in a more suitable environment, such as transplantation within the same species.

To ensure the clinical applicability of hSGOs, we performed allogeneic validation by generating mSGOs and verifying their effectiveness using allotransplantation. Culture of mSGOs in Matrigel with a modified medium revealed the formation of end buds and branches in the mSGOs. As end buds have been described as acinus during salivary gland development, our findings demonstrate a high degree of structural similarity between organoids and tissues [44]. The difference in differentiation capacity between mSGOs and hSGOs may be attributed to interspecies differences or to changes in stem cell quality, depending on the age of the patient tissue from which they were derived. Several studies have reported that stem cell functions, particularly regenerative capabilities, diminish with age [45, 46], and the efficiency of organoid formation in the stem cells obtained from aged mice is lower than that in those obtained from younger mice [47]. In our study, mSGOs were generated from 7-week-old mice, which is equivalent to the teenage years in humans. In contrast, hSGOs were established from salivary gland tissues obtained from patients with an average age of 58 ± 2.46 years. Consequently, mSGOs may possess superior stem cell proliferation and differentiation potential compared with hSGOs used in this study.

Following transplantation of EGFP-expressing mSGOs into a mouse model of xerostomia, damaged salivary glands exhibited regeneration accompanied by increased saliva secretion in the organoid-transplanted group. This indicates that salivary gland function was restored by mSGO transplantation. In summary, our study demonstrated that established mSGOs possess salivary gland properties and can lead to functional recovery. Given the effectiveness of mSGOs in allogeneic transplantation, our findings suggest the potential benefits of allogeneic transplantation in humans in the future.

## Conclusions

We developed a method for cultivating collagen-based hSGOs that exhibited self-renewal and differentiation capabilities comparable with those of Matrigel-based hSGOs. The regenerative potential of these collagen-based hSGOs was demonstrated by their successful engraftment in a mouse model of radiation-induced xerostomia. Notably, the expression of mucins, particularly MUC5B, in these transplanted organoids suggests an improvement in the salivary composition, highlighting their therapeutic potential. Furthermore, allotransplantation of mSGOs into a mouse model resulted in enhanced salivation, thereby validating their functional effectiveness. Our research has established a foundation for the future use of collagen-based hSGOs in allogeneic clinical trials.

### Electronic supplementary material

Below is the link to the electronic supplementary material.

**Supplementary Material 1: Supplementary Figure 1.** Single-cell RNA sequencing analysis of human submandibular gland tissue

**Supplementary Material 2: Supplementary table 1.** Age and gender distribution of patients in the study. **Supplementary table 2.** The components of culture medium for human and mouse salivary gland organoid

**Supplementary Material 3: Additional file 1.** Differentiated expressed gene (DEG) analysis for undifferentiated and differentiated hSGOs

**Supplementary Material 4: Additional file 2.** Gene expression data for human salivary gland tissue in scRNAseq analysis

**Supplementary Material 5: Additional file 3.** Gene expression data for human salivary gland organoids in scRNAseq analysis

## Data Availability

All data generated or analysed during this study are included in this published article and its supplementary information files. The datasets used and/or analysed during the current study are available from the corresponding author on reasonable request.

## Abbreviations

DM: Differentiation medium
ECM: Extracellular matrix
EM: Expansion medium
hSGO: Human salivary gland organoid
mSGO: Mouse salivary gland organoid
