# Supplementary material for: Salivary gland organoid transplantation as a therapeutic option for radiation-induced xerostomia
Source: Stem Cell Res Ther. 2024 Aug 26;15:265. doi: 10.1186/s13287-024-03833-x (PMC11346288; doi:10.1186/s13287-024-03833-x)
Supplement: Supplementary file 1 — Supplementary Material 1: Supplementary Figure 1. Single-cell RNA sequencing analysis of human submandibular gland tissue [file 13287_2024_3833_MOESM1_ESM.docx]

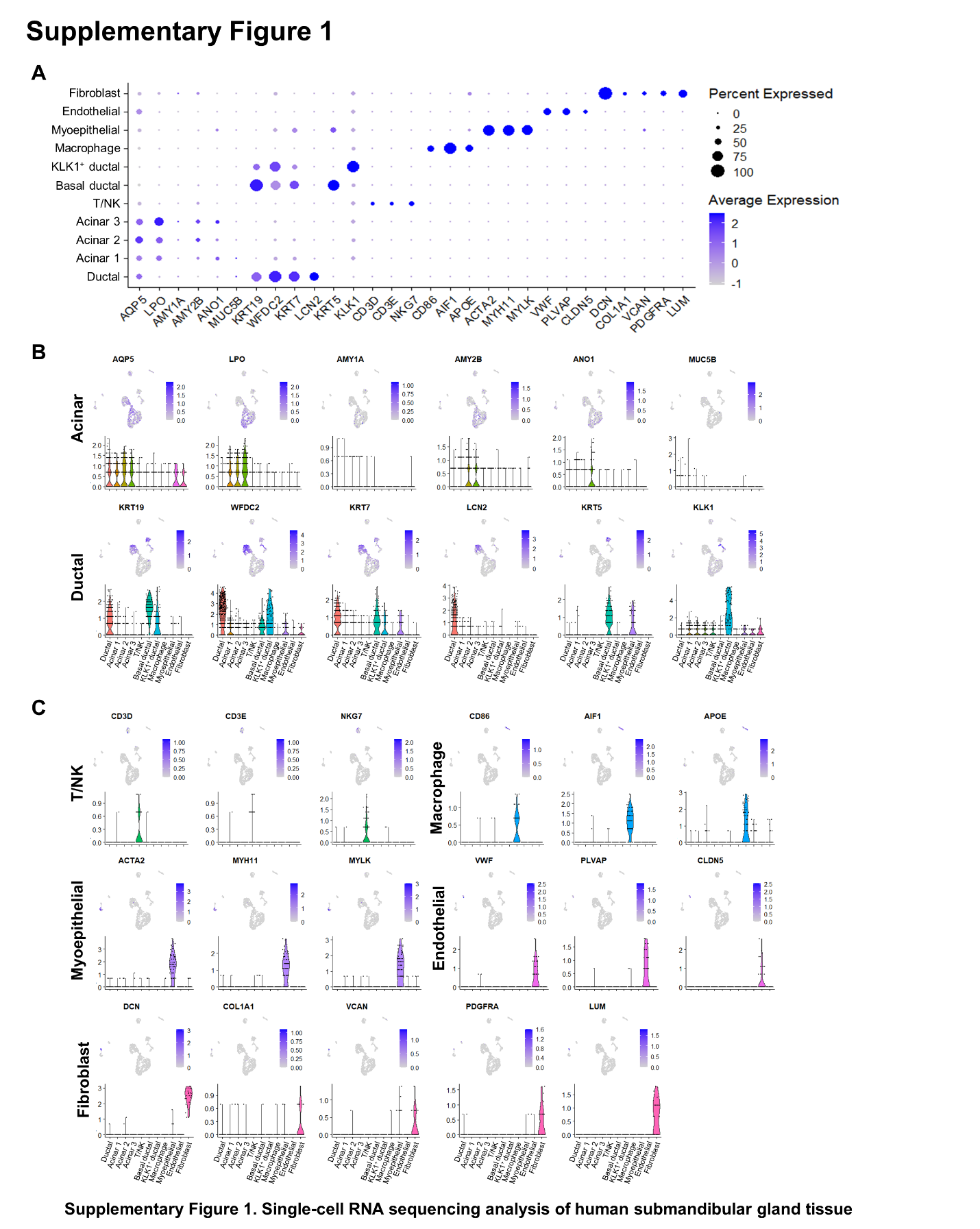


**Supplementary Figure 1. Single-cell RNA sequencing analysis of human submandibular gland tissue**

(A) Expression of marker genes for entire cell types and the corresponding clusters. (B) The UMAP and violin plot of the main population, acinar cells (*AQP5*, *LPO*, *AMY1A*, *AMY2B*, *ANO1*, and *MUC5B*) and ductal cells (*KRT19*, *WFDC2*, *KRT7*, *LCN2*, *KRT5*, and *KLK1*). (C) The UMAP and violin plot of the side population, T/NK cells (*CD3D*, *CD3E,* and *NKG7*), macrophages (*CD86*, *AIF1,* and *APOE*), myoepithelial cells (*ACTA2*, *MYH11*, and *MYLK*), endothelial cells (*VWF*, *PLVAP*, and *CLDN5*), and fibroblasts (*DCN*, *COL1A1*, *VCAN*, *PDGFRA*, and *LUM*).
