# Supplementary material for: Salivary gland organoid transplantation as a therapeutic option for radiation-induced xerostomia
Source: Stem Cell Res Ther. 2024 Aug 26;15:265. doi: 10.1186/s13287-024-03833-x (PMC11346288; doi:10.1186/s13287-024-03833-x)
Supplement: Supplementary file 2 — Supplementary Material 2: Supplementary table 1. Age and gender distribution of patients in the study. Supplementary table 2. The components of culture medium for human and mouse salivary gland organoid [file 13287_2024_3833_MOESM2_ESM.docx]

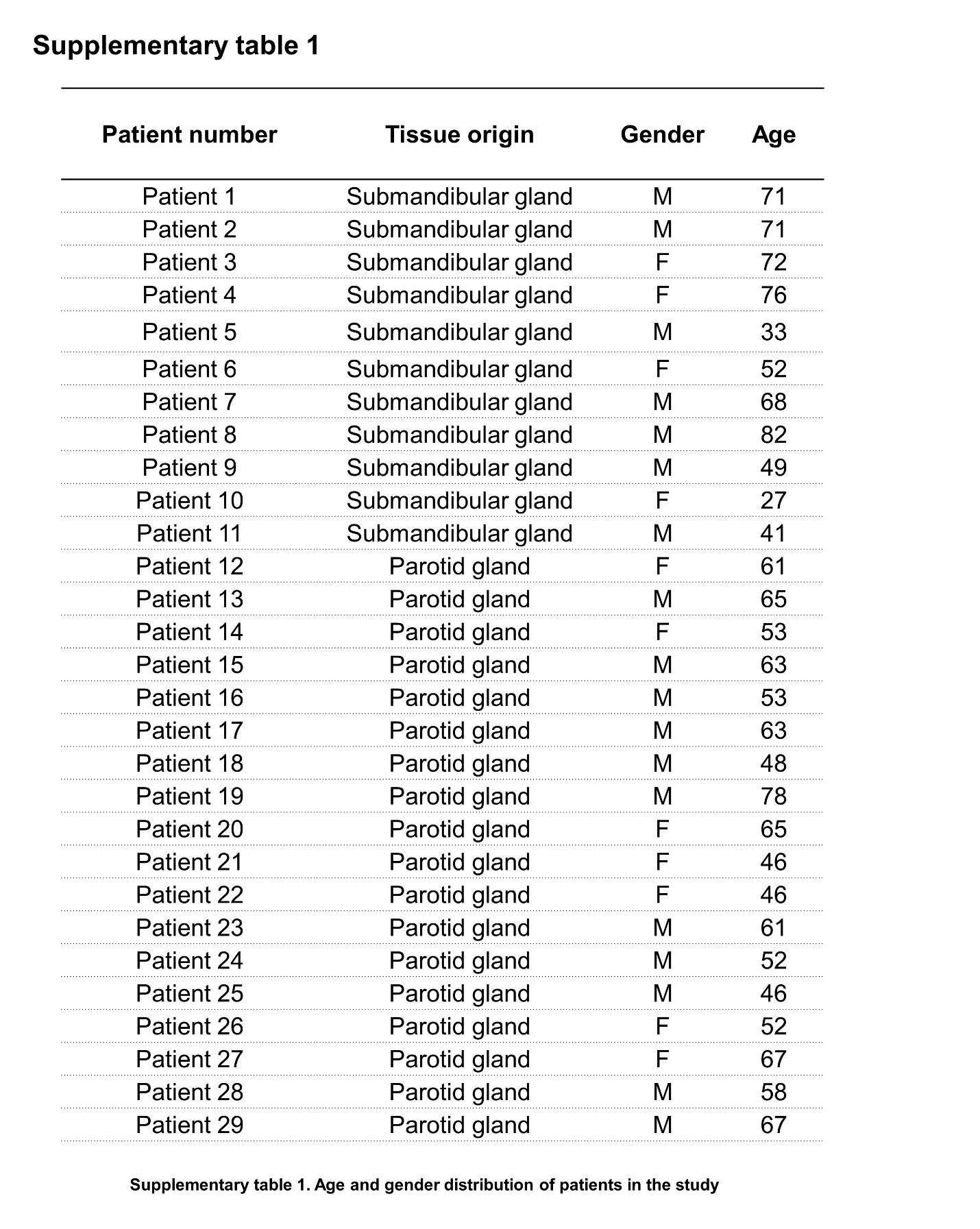


**Supplementary table 1. Age and gender distribution of patients in the study**


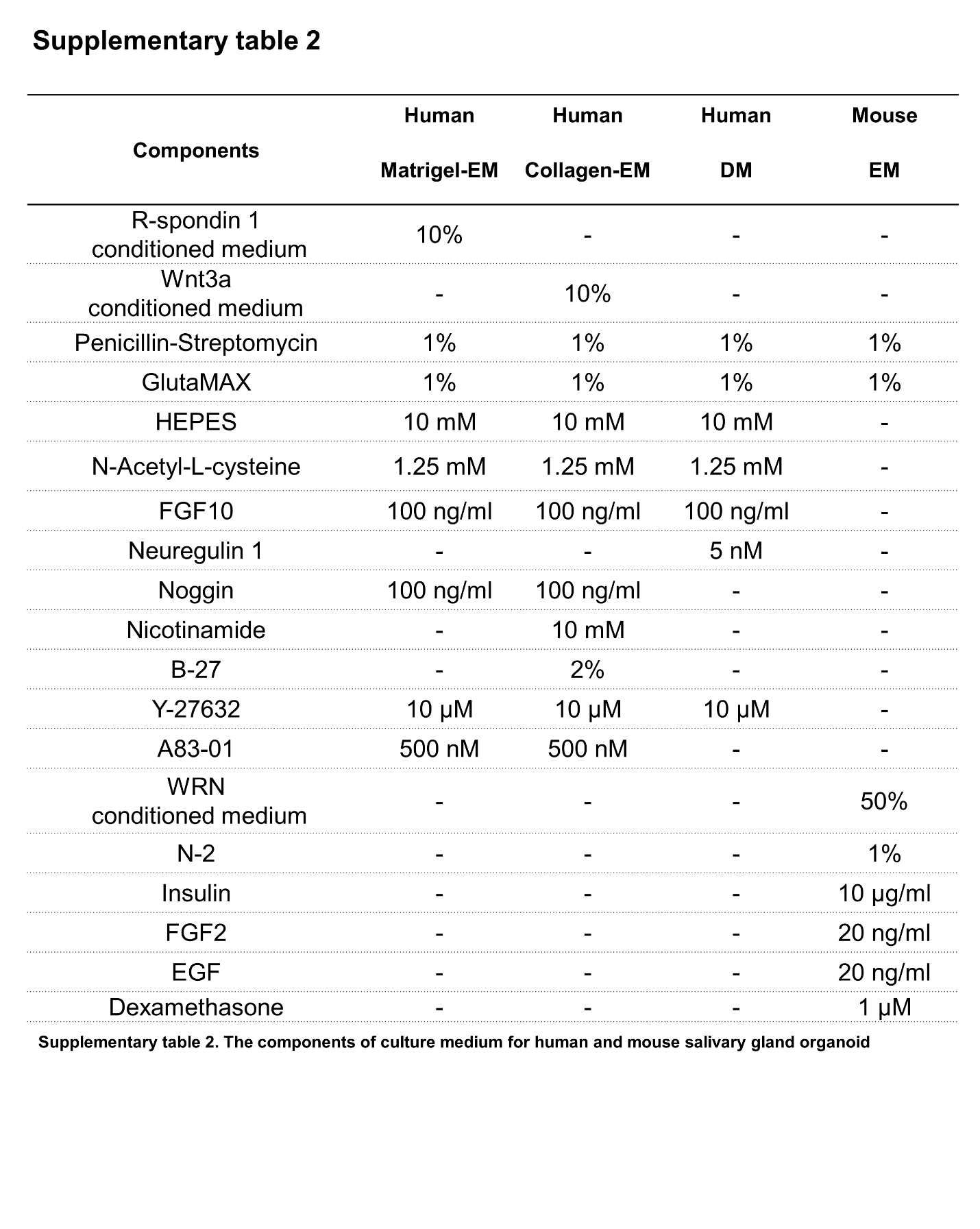


**Supplementary table 2. The components of culture medium for human and mouse salivary gland organoid** Abbreviations: DM (Differentiation medium), EM (Expansion medium), WRN (WNT, R-spondin and Noggin).
